# A Methodology for Using Large Language Models to Create User-Friendly Applications for Medicaid Redetermination and Other Social Services

**DOI:** 10.3389/ijph.2024.1607317

**Published:** 2024-08-16

**Authors:** Sumanth Ratna, William B. Weeks, Juan Lavista Ferres, Aneesh Chopra, Mayana Pereira

**Affiliations:** ^1^ Department of Computer Science, Yale University, New Haven, CT, United States; ^2^ Microsoft, AI for Good Lab, Redmond, WA, United States; ^3^ CareJourney, Arlington, VA, United States

**Keywords:** public health, access to health services, health service research, artificial intelligence (AI), Medicaid

## Background

Following the unwinding of Medicaid’s continuous enrollment provision, states must redetermine Medicaid eligibility, creating uncertainty about coverage [1] and the widespread administrative removal of beneficiaries from rolls [2].

Existing research demonstrates that Large Language Models (LLMs) can automate clinical trial eligibility query extraction [3], generation [4], and classification [5]. Given that Medicaid redetermination follows eligibility rules similar to those in clinical trials, we thought LLMs might help with Medicaid redetermination, as well.

Therefore, using the State of Washington, South Carolina, and North Dakota as examples, we applied LLMs to extract Medicaid rules from publicly available documents and transform those rules into a web application that could allow users to determine whether they are eligible for Medicaid. This paper describes the methodology we used.

## Methods

**FIGURE 1:**
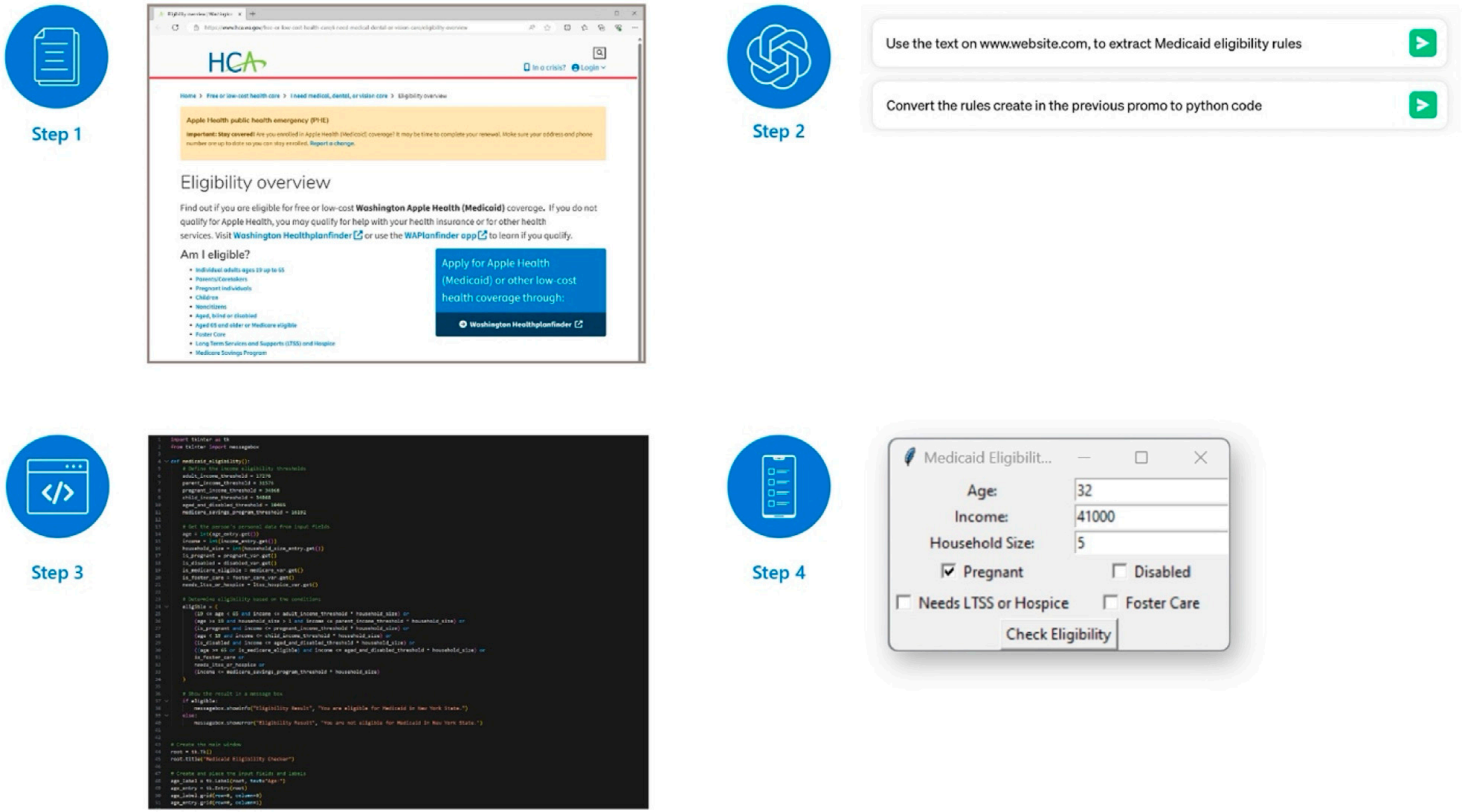
Diagram of our workflow (United States. 2024). The process that we used to develop an application starts with collecting documents that can be available as an HTML or other formats (Step 1). We used the text in the HTML as input to ChatGPT prompts; ChatGPT then extracted Medicaid rules from text and transformed rules into Python code (Step 2). The interaction with ChatGPT generated deployable Python code as output (Step 3) which, when deployed as an interactive application, can collect user information to determines Medicaid eligibility status (Step 4). (United States, 2024).

Using publicly available HyperText Markup Language (HTML) web pages that describe Medicaid eligibility rules as inputs to LLMs interactions, we used OpenAI GPT-4o and a rule extraction process to summarize those documents into rules related to eligibility criteria and to convert them into Python code which embedded them into a deployable application (Figure 1). We designed the application so that user-provided personal details trigger rules that determine eligibility status.

To demonstrate the generalizability of our pipeline, we studied three states: the State of Washington (where each Medicaid program has its own eligibility webpage), North Dakota (where a single webpage broadly describes eligibility for all Medicaid programs in a single section), and South Carolina (where a single webpage defines eligibility for each Medicaid program in order).

We evaluated the accuracy of the produced code by calculating the average time in minutes (over five attempts) that it took one of us (SR) to implement functional code across several scenarios. Functional code satisfies three properties: scope, meaning the program implements eligibility calculation for all Medicaid programs provided as input; accuracy, meaning the program’s eligibility calculations align with the natural language rules provided as input; and specificity, meaning the program identifies which specific Medicaid program the user is eligible for. We discretized the time needed to make each program “functional” by binning as follows: programs that required no modification received a score of 2; programs that required more than zero and less than 3 min to modify received a score of 1; and programs that needed 3 min or longer received a score of 0. As such, higher scores correspond to higher-quality LLM outputs, while lower scores correspond to lower-quality LLM outputs.

We also studied the effects of two programmer-set parameters on results. The first was temperature, which controls the amount of randomness in the output, with temperature 0.0 generating more deterministic responses and temperature 1.0 generating more random responses. The second was “top p,” which controls how much responses can deviate from the input’s topic, with “top p” 1.0 generating more creative responses and “top p” 0.0 generating responses that are more restrictive and do not elaborate on defined criteria.

For North Dakota and South Carolina, we used GPT-4o in a two-step pipeline: first, to extract eligibility rules (in natural language); second, to define eligibility rules for public consumption, using Python 3.

For the State of Washington, we introduced another variable into the pipeline; we varied the order of rule extraction: one method converted input to rules for each Medicaid category, then concatenated eligibility rule guidelines, then asked GPT-4o to write Python code to implement those guidelines (“Combine to Python”); the other method converted input to Python directly, concatenated the Python snippets, and asked GPT-4o to combine the snippets (“Python to Combine”). We calculated the cost to run our pipeline according to OpenAI’s pricing model, which is publicly available.

Our study used publicly available data and was exempt from human subjects’ review.

## Results

For both North Dakota and South Carolina, we found optimal results at a temperature of 0.5 and a “top p” of 0.0 (Table 1). This aligned with our expectations, as we anticipated a moderate temperature would allow creativity in code-generation while maintaining accuracy.

**TABLE 1:** Experiment results for North Dakota and South Carolina. (North Dakota and South Carolina, United States, 2024).

		North Dakota			South Carolina		
		Temperature value			Temperature value		
		0.0	0.5	1.0	0.0	0.5	1.0
“top p” value	**1.0**	1.2	1.2	2.0	1.2	1.6	1.4
	**0.5**	1.4	1.4	2.0	1.6	1.6	1.0
	**0.0**	2.0	**2.0**	1.8	1.8	**2.0**	1.6

The values show the average of the binned scores across five efforts, where higher scores correspond to fewer number of minutes needed to modify the resultant Python code into a functional and accurate application for end users. We show results across several values of temperature (which controls the amount of randomness in the output) and “top p” (which controls how much responses can deviate from the input’s topic). For each state, the optimal result is in bold.

For the State of Washington, we found that there was no significant difference between “Combine to Python” and “Python to Combine.” We found that moderate temperature (at a value of 0.5) and “top p” (at a value of 0.5) produced high-quality implementations that were specific and required no human corrections (Table 2).

**TABLE 2:** Experiment results for the State of Washington. (State of Washington, United States, 2024).

		“Combine to Python”			“Python to combine”		
		Temperature value			Temperature value		
		0.0	0.5	1.0	0.0	0.5	1.0
“top p” value	**1.0**	1.2	1.4	1.0	1.0	1.4	1.0
	**0.5**	1.2	1.0	1.4	1.2	1.2	1.4
	**0.0**	1.4	**1.8**	1.4	1.4	**1.8**	1.0

The values show the average of the binned scores across five efforts, where higher scores correspond to fewer number of minutes needed to modify the resultant Python code into a functional and accurate application for end users. We show results across several values of temperature (which controls the amount of randomness in the output) and “top p” (which controls how much responses can deviate from the input’s topic). The optimal result for each approach is in bold.

The cost for North Dakota was approximately $0.07 per experiment (specific top p and temperature combination); that for South Carolina was approximately $0.17, and that for the State of Washington was approximately $0.23 per experiment.

## Discussion

For three states, we used publicly available information on Medicaid eligibility criteria and OpenAI GPT-4o to generate Python code that created interactive applications to help determine Medicaid eligibility. We could do so relatively easily and in a replicable way that could improve service delivery efficiency, potentially while reducing errors caused by manual processing. Our pipeline performed well across all three states, suggesting that our methods are generalizable, and was inexpensive.

Overall, the methodology that we describe could easily be used by states to develop easily understood guidance for potential beneficiaries of a variety of state-run programs. To ensure the reliability and trustworthiness of the system, states implementing this process should address the limitations of LLMs, including potential inaccuracies and fabricated content; further, should they choose to use this method, states should follow ethical guidelines and proper procedures for its deployment and should rigorously verify the accuracy of application in determining Medicaid eligibility.

This general framework might be applicable to multiple government eligibility processes, and, if applied widely, could result in services that are more accessible, transparent, and efficient than traditional methods. The methodology facilitates development of applications in several languages, significantly impacting beneficiaries with limited English proficiency, who are 5.3 times more likely to lose Medicaid benefits than English-proficient ones [6]. At virtually no cost and with little effort, a process like the one described here might be used to integrate LLMs into healthcare decision support [7], ease the burden of individuals navigating bureaucratic processes in a variety of social services settings, and foster equitable access to health and other benefits.
